# Combinatory Treatment with Oseltamivir and Itraconazole Targeting Both Virus and Host Factors in Influenza A Virus Infection

**DOI:** 10.3390/v12070703

**Published:** 2020-06-29

**Authors:** Sebastian Schloer, Jonas Goretzko, Stephan Pleschka, Stephan Ludwig, Ursula Rescher

**Affiliations:** 1 Institute of Medical Biochemistry, Center for Molecular Biology of Inflammation and “Cells in Motion” Interfaculty Centre, University of Muenster, Von-Esmarch-Str. 56, D-48149 Muenster, Germany; SebastianMaximilian.Schloer@ukmuenster.de (S.S.); Jonas.Goretzko@ukmuenster.de (J.G.); 2 Institute of Medical Virology, Justus-Liebig-Universität, Schuberststr. 81, D-35392 Gießen, Germany; Stephan.Pleschka@viro.med.uni-giessen.de; 3 Institute of Virology (IVM) and “Cells in Motion” Interfaculty Centre, University of Muenster, Von-Esmarch-Str. 56, D-48149 Muenster, Germany; ludwigs@uni-muenster.de

**Keywords:** influenza A virus, oseltamivir, itraconazole, host-directed therapy, drug repurposing, combination therapy, additivity

## Abstract

Influenza virus infections and their associated morbidity and mortality are a major threat to global health. Vaccination is an effective influenza prevention measure; however, the effectiveness is challenged by the rapid changes in the influenza virus genome leading to viral adaptation. Emerging viral resistance to the neuraminidase inhibitor oseltamivir limits the treatment of acute influenza infections. Targeting influenza virus-host interactions is a new and emerging field, and therapies based on the combination of virus- and host-directed drugs might significantly improve treatment success. We therefore assessed the combined treatment with oseltamivir and the repurposed antifungal drug itraconazole on infection of polarized broncho-epithelial Calu-3 cells with pdm09 or Panama influenza A virus strains. We detected significantly stronger antiviral activities in the combined treatment compared to monotherapy with oseltamivir, permitting lower concentrations of the drug than required for the single treatments. Bliss independence drug interaction analysis indicated that both drugs acted independently of each other. The additional antiviral effect of itraconazole might safeguard patients infected with influenza virus strains with heightened oseltamivir resistance.

## 1. Introduction

With 3–5 million severe cases worldwide and 300,000–700,000 fatalities annually, influenza virus is not only a major threat to public health, but also poses a substantial burden on health care resources. In addition to the annual epidemics, sudden and unpredictable pandemics present global health emergencies. Vaccination is a very effective measure that has been available for more than six decades [1]. However, the high mutation rate of the viral genome causing continuous changes (antigenic drift) requires a constant update of the vaccines [2,3,4]. Moreover, vaccination does not protect against outbreaks caused by the emergence of new viral subtypes with new surface glycoproteins due to genome reassortment between two different strains (antigenic shift) [5]. Thus, antiviral drugs are essential additions for effective infection control.

To initiate the infection process, the hemagglutinin (HA) protruding from the envelope of influenza A virus (IAV) attaches to the host cell via binding to sialic acid moieties on the cell surface, leading to the endocytosis of the virion [6,7]. The pH drop in late endosomes (LE) elicits HA-mediated fusion of the virus envelope with the endosomal membrane, leading to the subsequent release of the viral genome into the host cell cytosol [8,9]. To avoid the rebinding of budding progeny viruses, the IAV surface protein neuraminidase (NA) cleaves the glycosidic linkages of terminal neuraminic acid residues, thus ensuring virus release and spread [7]. Because of the vital functions of sialic acid residues in the IAV infection process, the viral NA constitutes an attractive target for anti-IAV therapy [10,11]. The NA active site is conserved among IAV and influenza B viruses (IBV), and, therefore, sialic acid analogs as competitive NA inhibitors were already developed in the 1990s. The most commonly used NA inhibitor is oseltamivir; however, the constant risk of emerging resistance to this drug became evident in 2007/2008 when more than 90% of circulating seasonal H1N1 strains in the northern hemisphere acquired a resistance mutation and became insensitive to oseltamivir [11,12]. The pandemic strain H1N1pdm09 (pdm09), which replaced the old H1N1 lineage in 2009, fortunately did not carry this resistance mutation; therefore, NA inhibitors such as oseltamivir are still recommended for influenza treatment [13,14,15]. However, the emergence of oseltamivir-resistant IAV and IBV strains has been frequently reported in the last decade [12]. A novel endonuclease inhibitor for treatment of influenza A and influenza B infection, baloxavir marboxil [16], was recently approved for medical use in Japan and the United States. While baloxavir marboxil is another success in the development of antiviral medication, especially because of the approval extension for treatment of high-risk patients, oseltamivir is still the leading, most commonly used drug in influenza infection treatment. The second class of approved antivirals are the adamantanes amantadine and rimantadine, which both inhibit the IAV proton channel M2. Nevertheless, due to high resistance rates, especially among the most common, globally circulating H1N1 and H3N2 subtypes, the adamantanes are not recommended anymore for prophylaxis and treatment [17,18].

The ability of IAV to rapidly develop resistance against antivirals underscores the need for novel therapeutic options aside from direct targeting of the virus. The use of host-directed therapeutics which interfere with host cell factors that are essentially required for a successful replication process is an emerging approach to counteract viral infection [19,20,21,22,23]. Because the pathogens critically depend on the host cell, an escape from host-directed therapy (HDT) would require substantial changes in the infection cycle and is, therefore, much less likely to occur [20,21]. In previous studies, we concentrated on the crucial role of host cell cholesterol homeostasis in the IAV infection cycle. Indeed, we could identify the late endosomal cholesterol balance as a druggable host cell target for antiviral intervention [24,25,26]. Moreover, the antifungal triazole derivate drug itraconazole, which blocks the LE cholesterol export via inhibition of the late endosomal cholesterol transport protein Niemann-Pick C1 (NPC1), displayed antiviral capability against various IAV and IBV subtypes in vitro and in a preclinical murine model of IAV infection [24].

Combination therapy, i.e., the treatment of influenza virus infection with at least two drugs with different modes of action, is considered a promising strategy for circumventing therapy resistance, based on the notion that the virus will stay susceptible to at least one of the drugs. A wide number of studies have investigated the combinatory use of antivirals belonging to the adamantanes and NA inhibitors [27,28,29]. In this study, we explored the effect of a combinatory use of the antiviral drug oseltamivir and the host-directed anti-infective drug itraconazole on IAV infection in a cell culture model of polarized bronchial cell monolayers. Combinations of oseltamivir and itraconazole exerted significantly stronger antiviral activities compared to the respective monotherapies, and lower oseltamivir concentrations were required. Assessment of the mode of action indicated that the combination of both drugs showed additivity. The additional antiviral effect of itraconazole might safeguard patients infected with influenza strains with heightened oseltamivir resistance.

## 2. Materials and Methods 

### 2.1. Viruses and Plaque Titration

The influenza virus isolates A/Hamburg/04/2009 (H1N1)pdm09 and A/Panama/2007/99 (H3N2) were taken from the strain collection of the Institute of Virology, University of Münster, Germany. Both strains were amplified on MDCK cells. Viral titers were determined via standard plaque assays in MDCK cells [26].

### 2.2. Cells

The human bronchial epithelial cell lines Calu-3 and the Madin-Darby canine kidney (MDCK) II cells were cultivated in Dulbecco’s modified Eagle’s medium (DMEM) with 10% standardized fetal bovine serum (FBS Superior; Merck, Darmstadt, Germany), 2 mM L-glutamine, 100 U/mL penicillin, 0.1 mg/mL streptomycin, and 1% non-essential amino acids (Merck, Darmstadt, Germany) in a humidified incubator at 5% CO2 and 37 °C. Polarized Calu-3 monolayers were established on semipermeable supports with a 0.4 µm pore size (12-well format, Corning, New York, N.Y., USA) for 4 days. The transepithelial electrical resistance (TEER) of the confluent monolayers was measured with a Millicell-ERS (Electrical Resistance System) voltohmmeter (Merck Millipore, Darmstadt, Germany) and reached around 800 Ωcm^2^. To assess polarization on the cytomorphological level, polarized cells grown to confluency were evaluated for the distribution of the tight junction-associated protein Zonula occludens-1 (ZO-1) as a marker for the apical surface, and the basolateral marker β-catenin. In brief, cells were fixed with 4% paraformaldehyde and stained with anti-ZO-1 antibodies (rabbit polyclonal, Invitrogen, Waltham, M.S., USA) and anti-β-catenin antibodies (mouse monoclonal, BD Transduction Laboratories, Heidelberg, Germany). Nuclei were visualized with DAPI (300 nM). Z-stacks (11 focal planes/stack) were acquired on an LSM 800 confocal microscope (Carl Zeiss, Jena, Germany equipped with a Plan-Apochromat 63×/1.4 oil immersion objective. Images were analyzed with Imaris microscopy image analysis software (Oxford Instruments, Zurich, Switzerland).

### 2.3. Cytotoxicity Assay

Calu-3 cells were cultured at the indicated concentrations with either DMSO as the solvent, oseltamivir or itraconazole for 24 h. Subsequently, MTT 3-(4,5-dimethylthiazol-2-yl)-2,5-diphenyltetrazolium bromide was added for 4 h, and cell viability was evaluated via OD562 measurements according to the manufacturer’s protocols (Sigma, Darmstadt, Germany). The microbial alkaloid staurosporine (1 µM, Sigma, Darmstadt, Germany) was used to induce cell death.

### 2.4. Inoculation of Polarized Calu-3 Monolayers and Drug Treatment

For infection, the monolayers were washed with PBS and were inoculated at a multiplicity of infection (MOI) of 0.1 of virus diluted in infection-PBS (PBS containing 0.2% bovine serum albumin (BSA), 1 mM MgCl_2_, 0.9 mM CaCl2, 100 U/mL penicillin and 0.1 mg/mL streptomycin, 300 µL/well) at 37 °C for 30 min. Subsequently, cells were washed with PBS and further cultured in infection-DMEM (serum-free DMEM containing 0.2% BSA, 1 mM MgCl_2_, 0.9 mM CaCl_2_, 100 U/mL penicillin, and 0.1 mg/mL streptomycin) at 5% CO2 and 37 °C. *N*-*p*-tosyl-ʟ-phenylalanine chloromethyl ketone (TPCK)-treated trypsin (Sigma-Aldrich, Darmstadt, Germany, 1 μg/mL) was added to facilitate infection. Cells were treated with itraconazole (Sigma, Darmstadt, Germany, solubilized in DMSO at 2 mg/mL) and/or oseltamivir (Sigma, Darmstadt, Germany, solubilized in H_2_O, 10 mg/mL) 2 h post-infection (p.i.). Aliquots of apical culture supernatants were collected at 24 h p.i. and were immediately frozen at −80 °C until titration. To assess antiviral activities, plaque-forming units (PFU) in the cell culture supernatants were expressed as percentages of PFU in supernatants of control cells treated with solvent only which represented maximum infection.

### 2.5. Data Analysis

A priori power analysis (G*Power 3.1, [30]) was used to estimate the required sample sizes and post hoc analysis confirmed that powers > 0.99 were achieved. Data were analyzed with Prism 8.00 (GraphPad, San diego, C.A., USA). For dose-response curves, drug concentrations were log-transformed and virus titers were expressed as percentages of the mean virus titer in control cells (treated with the solvent DMSO). For assessment of inhibition, the reduction of viral titers in treated cells was expressed as percentages of titers found in control cells. For the determination of logIC_50_ values, data were analyzed by fitting a curve using nonlinear regression and the 4 parameter logistic model with a standard Hill slope of 1.0. To detect a potentially global impact of the combination on the respective individual antiviral drug activity, the best-fit values of the unshared logIC_50_ values were compared by extra sum-of-squares F tests. Significant reductions in virus titers compared to control cells were detected by one-way ANOVA followed by Dunnett’s multiple comparisons test. To analyze the significance of itraconazole-mediated reduction at a fixed oseltamivir concentration, the reduction in virus titer was compared to the respective oseltamivir-only sample by two-way ANOVA followed by Dunnett’s multiple comparisons test. *p* < 0.05 was considered significant. Drug combinatory effects were assessed based on the Bliss independence model by calculating the difference ΔE = E_expected_ − E_observed_, with E_expected_ = E_O_ + E_I_ − E_O_ × E_I_. E_O_, effect of oseltamivir at a given concentration, E_I_, effect of itraconazole at a given concentration, E_observed_, effect of combined treatment at given concentrations. ΔE > 0 indicates antagonism, ΔE < 0 indicates synergism. SynergyFinder, an open-source free stand-alone web application for the analysis of drug combination data [31], was used to evaluate synergy based on the Loewe additivity, and Zero Interaction Potency (ZIP) reference models.

## 3. Results

**Figure 1:**
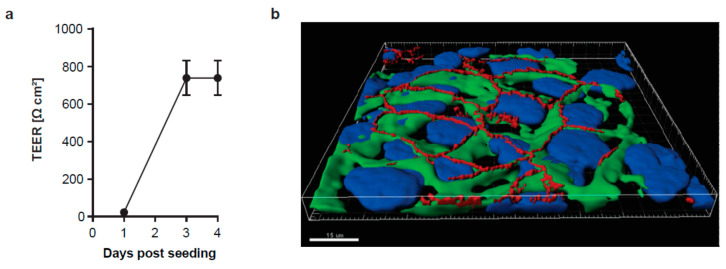
Establishment of polarized monolayers of the airway epithelial cell line Calu-3 cultured on semipermeable supports. (**a**) Transepithelial electrical resistance (TEER) was measured over the 4 days culture period. (**b**) 3D confocal microscopy image of polarized Calu-3 cells. To evaluate cell differentiation and polarization, the tight junction-associated protein ZO-1 (red signal) at the apical surface and the basolateral marker β-catenin (green signal) were immunostained. Nuclei were visualized with DAPI. Note the honeycomb-like ZO-1 pattern. Scale bar, 15 µm.

The majority of the most common circulating IAV subtypes already acquired resistance against the approved antiviral drug amantadine and there is increasing evidence that the commonly used drug oseltamivir will sooner or later share this fate. To analyze the effect of a combination treatment with oseltamivir and the host-targeting drug itraconazole, we selected two representatives of the currently circulating H1N1 and H3N2 subtypes, namely a strain of the 2009 pandemic IAV H1N1 viruses (pdm09), which has become the dominant H1N1 lineage and is still oseltamivir-sensitive, and the human seasonal H3N2 isolate A/Panama/2007/99 (Panama) [14,15]. We utilized the human bronchial epithelial cell line Calu-3 cultured submerged in media on semipermeable supports as an in vitro infection model. After four days of cultivation, the adherent monolayer typically reached a transepithelial electrical resistance of 800 Ωcm^2^ (Figure 1a), and the tight junction-associated protein Zona occludens-1 (ZO-1) was targeted to the apical surface in a cobblestone (honeycomb) pattern (Figure 1b), indicating successful apicobasal polarization of the confluent epithelial monolayers. 

**Figure 2:**
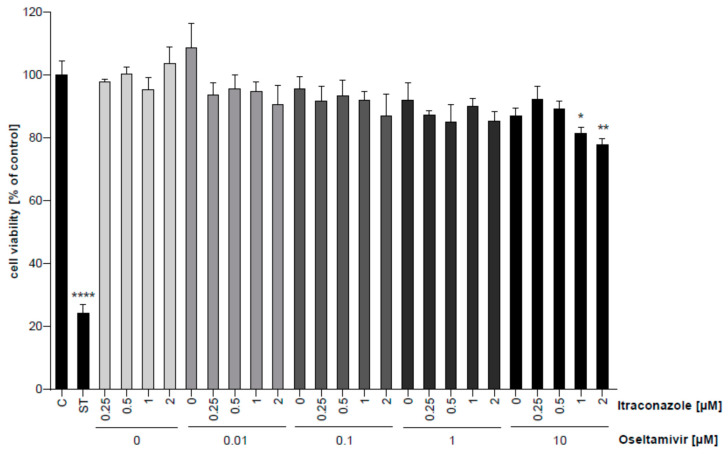
Analysis of the cytotoxicity of oseltamivir and itraconazole in single or combinatory treatment. Polarized Calu-3 monolayers were treated at the indicated concentrations with oseltamivir, itraconazole, or combinations for 24 h. Data represent mean percentages of viable cells ± SEM, with mean viability in control cells treated with the solvent DMSO (C) set to 100%. Cell death induced by staurosporine (ST) served as a positive control. *n* = 3, one-way ANOVA followed by Dunnett’s multiple comparison test. * *p* < 0.05, ** *p* < 0.01, **** *p* < 0.0001.

We first tested whether treatment with any of the drugs—single or combined—affected cell survival. In line with their clinical use, neither oseltamivir nor itraconazole alone had an impact on cell viability. The combination treatments were also well-tolerated, and a notable reduction (25–30%) in cell viability was only seen when cells were simultaneously treated with the highest concentrations of oseltamivir (10 µM) and high doses of itraconazole (Figure 2).

**Figure 3:**
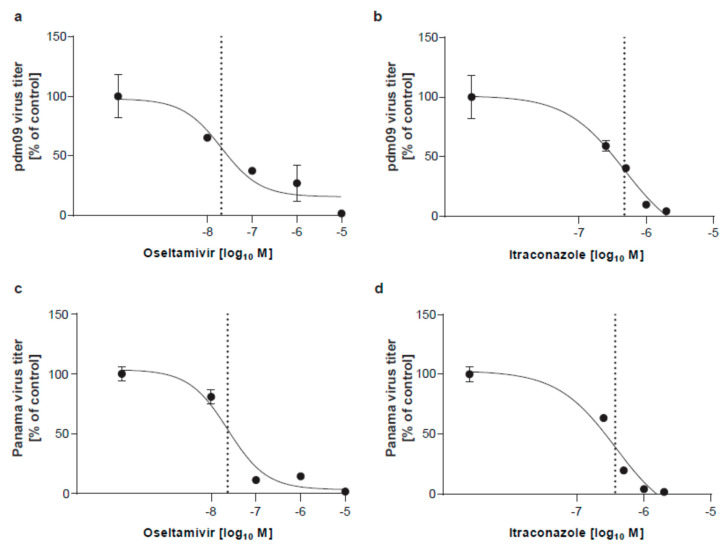
Analysis of the anti-influenza A virus (IAV) activity of oseltamivir and itraconazole. Polarized Calu-3 monolayers were infected with 0.1 MOI of (**a**,**b**) IAV H1N1pdm09 (pdm09) or (**c**,**d**) A/Panama/2007/99 (Panama) for 24 h. At 2 h p.i. cells were treated with oseltamivir (**a**,**c**) or itraconazole (**b**,**d**) at the indicated concentrations for 16 h. Data represent mean percentages of viral titers ± SEM, with mean virus titer in control cells (treated with the solvent DMSO) set to 100%; *n* = 3. LogIC_50_ values were determined by fitting a non-linear regression model.

**Figure 4:**
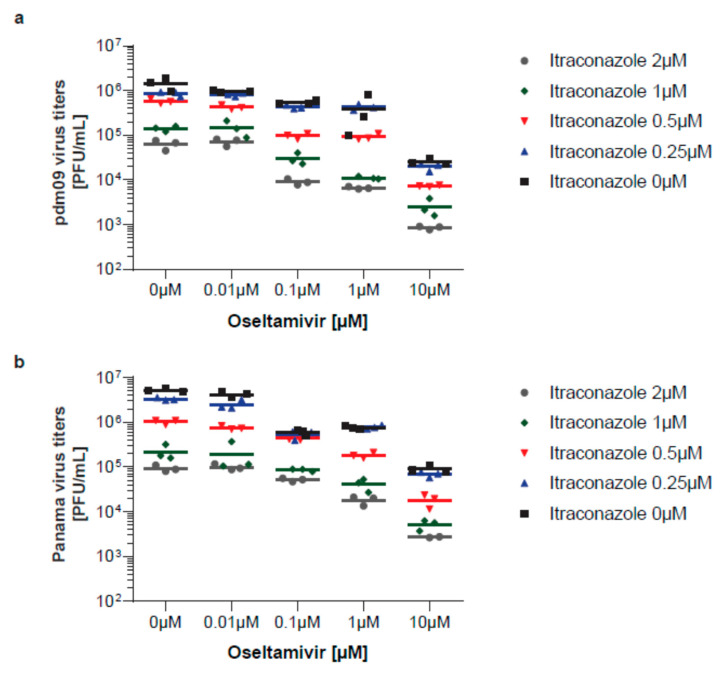
Individual virus titers obtained in all experimental samples. Viral titers obtained in cells infected with (**a**) pdm09 and (**b**) Panama are expressed as plaque-forming units (PFU) per mL with means and are presented on a logarithmic scale.

To confirm the anti-IAV activity of both drugs, the polarized monolayers were inoculated with 0.1 MOI of either pdm09 or Panama isolate, and the drugs were added 2 h p.i. Both IAV subtypes replicated with high efficiency in control cells treated with solvent only. As shown in Figure 3, both drugs displayed significant antiviral activity for both strains over the range of concentrations tested (see also Figure 4). Nonlinear fitting of the concentration-response curves determined EC_50_ values for the single-drug treatments. As shown in Figure 3, the half-maximum effective concentration of oseltamivir was reached at 20.56 nM (pdm09) and 23.41 nM (Panama), respectively. Itraconazole EC_50_ was 0.47 µM for pdm09 and 0.37 µM for Panama, well in accordance with our previous results of EC_50_ value of 0.57 µg/mL, determined for itraconazole-treated A549 cells infected with the Münster variant of the H1N1 influenza virus A/Puerto Rico/8/34 (PR8) [24].

Next, we screened treatments with oseltamivir/itraconazole combinations for their antiviral activities. The actual virus titers are presented in Figure 4. 

**Figure 5:**
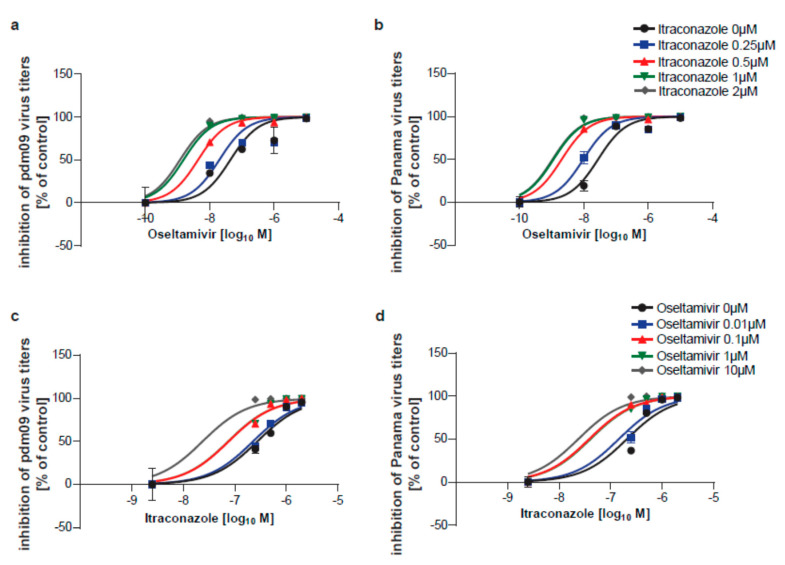
Analysis of the anti-IAV activity of oseltamivir and itraconazole in combinatory treatments. Polarized Calu-3 monolayers were infected with 0.1 MOI of (**a**,**c**) pdm09 or (**b**,**d**) Panama for 24 h. At 2 h p.i. cells were treated with combinations of oseltamivir and itraconazole at the indicated concentrations for 16 h. Data represent mean percentages ± SEM of inhibition, with mean virus titer in control cells (treated with the solvent DMSO) set to 100%; *n* = 3. LogEC_50_ values were determined by fitting a non-linear regression model and were compared for shared logEC_50_ values.

We first compared the half-maximum effective concentration of both drugs in combination (Figure 5). For both virus subtypes, oseltamivir logEC_50_ values were shifted toward lower concentrations required to reach EC_50_ in the presence of itraconazole, and similarly shifted logEC_50_ values were also found for itraconazole in combination with oseltamivir.

**Figure 6:**
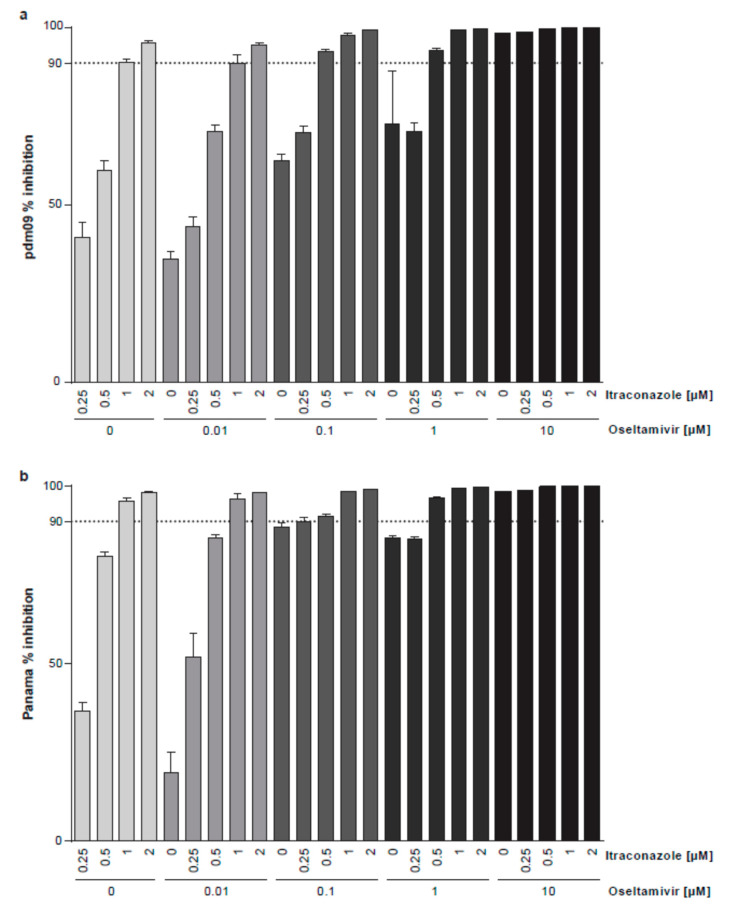
Analysis of the anti-IAV activity of combination treatment with oseltamivir and itraconazole. Polarized Calu-3 monolayers were infected with 0.1 MOI of (**a**) pdm09 or (**b**) Panama for 24 h. Oseltamivir and itraconazole were added at the indicated combinations 2 h p.i. Mean percentages of reduction in viral titers ± SEM, with mean virus titer in control cells (treated with the solvent DMSO) set to 100%; *n* = 3. Dotted line, 90% reduction in viral titer.

Generally, virus titers were potently reduced for all treatments and both virus subtypes. Individually applied, 10 µM oseltamivir was required to achieve ≥90% reduction of maximum virus titers produced in control cells, and 1 µM itraconazole was already sufficient to yield a similar reduction (Figure 6). Combinations of 0.5 µM itraconazole with either 0.1 or 1 µM oseltamivir had an inhibitory effect of ≥90%, which was not seen when the drugs were given individually.

**Figure 7:**
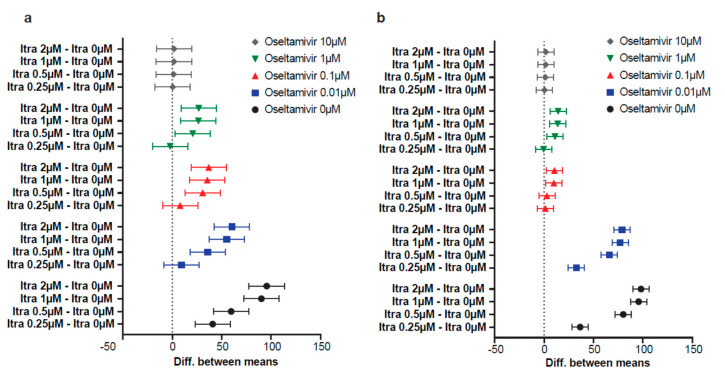
Analysis of the anti-IAV activity of combination treatments with oseltamivir and itraconazole. Polarized Calu-3 monolayers were infected with 0.1 MOI of (**a**) pdm09 or (**b**) Panama for 24 h. Oseltamivir and itraconazole were added at the indicated combinations 2 h p.i. For each oseltamivir concentration, mean percentages of reduction in viral titers ± SEM were compared to the respective oseltamivir treatment without itraconazole. Data are expressed as differences and 95% confidence intervals of the comparisons.

Computation of the 95% confidence intervals of the differences between combinations and the respective single dose of oseltamivir revealed that itraconazole increased the antiviral activity in the range of 0–1 µM oseltamivir; however, the differences were less pronounced with increasing concentrations of oseltamivir (Figure 7).

**Figure 8:**
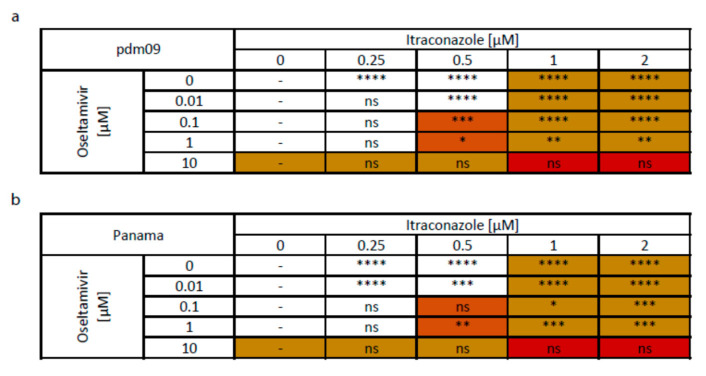
Overview of anti-IAV activities of combination treatments with oseltamivir and itraconazole. Polarized Calu-3 monolayers were infected with 0.1 MOI of (**a**) pdm09 or (**b**) Panama for 24 h. Oseltamivir and itraconazole were added at the indicated combinations 2 h p.i. Colored cells indicate ≥90% significant reduction in viral titers. Combinations with higher inhibitory activities than the respective individual drug concentrations are highlighted in yellow. Orange cells indicate inhibition ≥90% for combinations, with the individual drug treatment reaching inhibition ≤90%. Red cells indicate combinations leading to significant cytotoxicity. Asterisks indicate significant differences in antiviral activities when the reduction in viral titers found with the combined treatments was compared to the respective oseltamivir concentration.

Figure 8 summarizes the effects of combined treatments. Inhibition of at least 90% could be achieved with either oseltamivir and itraconazole single treatment. Combination treatments, except for the 10 µM oseltamivir treatment, the highest concentration used, tended to yield better inhibitory effects, and ≥90% inhibition already could be reached when 0.5 µM itraconazole was added to 0.1 µM oseltamivir (in case of pdm09) or when cells were treated with 1 µM oseltamivir (for both strains).

**Figure 9:**
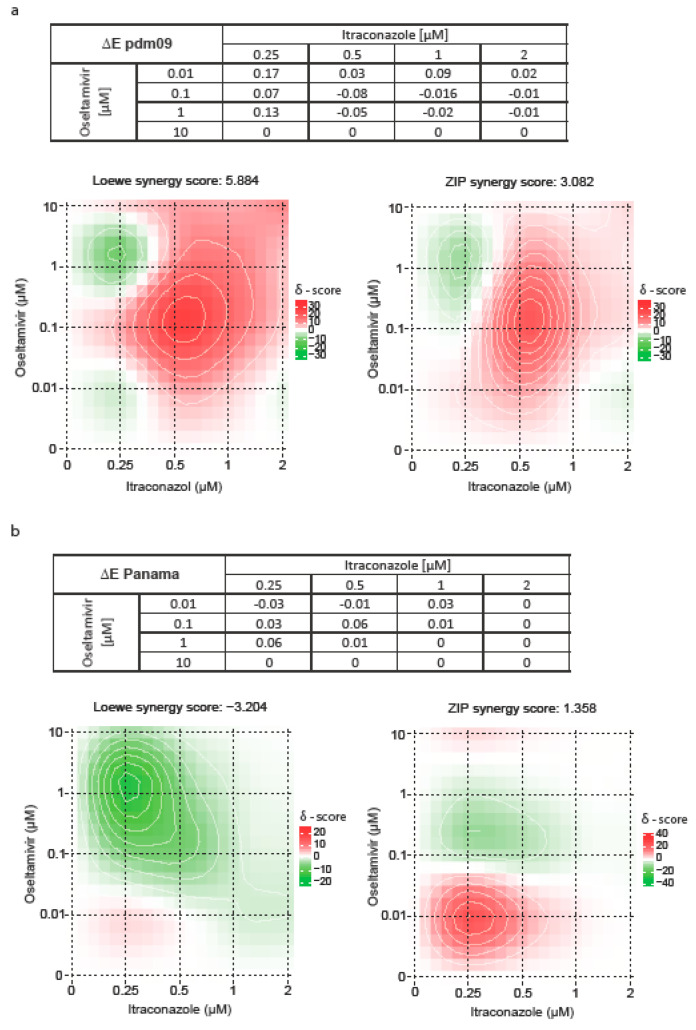
Assessment of synergistic effects of combined treatment with oseltamivir and itraconazole. Bliss independence, Loewe additivity and ZIP reference models were used to assess drug interaction profiles for (**a**) pdm09 and (**b**) Panama virus infections. Bliss independence model, ΔE < 0 indicates synergism, ΔE > 0 indicates antagonism. Loewe and ZIP synergy scores, color scale bars indicate mode and strength of interaction.

The enhanced antiviral activities seen in the combinatory treatments could result from independent, additive drug responses or from synergistic effects. We therefore applied several commonly used reference models to evaluate drug combination data. The widely used Bliss independence model compares the expected with the observed effects and is based on the assumption that the respective drugs affect independent pathways. Unlike Bliss independence, the Loewe additivity model requires dose-response curves for the monotherapies and the assumption is a shared mechanism of action. The ZIP reference model compares the change in the potency between the monotherapy and the combination dose-response curves. The drug interaction analyses, summarized in Figure 9, resulted in consistent patterns across the models. For infection with pdm09, 0.25 µM itraconazole tended to antagonize oseltamivir antiviral action, whereas higher itraconazole doses were synergistic, with an optimum at 0.5 µM itraconazole/0.1 µM oseltamivir. For Panama-infected cells, only the drug combinations 0.01 µM oseltamivir/0.25–0.5 µM itraconazole showed synergistic actions. However, all of the observed effects were not pronounced, arguing for rather additive effects.

## 4. Discussion

We analyzed the antiviral effects of combined treatment with the IAV neuraminidase-targeting drug oseltamivir and itraconazole, a licensed antifungal that we previously reported to efficiently block IAV endosomal escape in cell culture [24] on the replication efficiency of two different IAV strains. Itraconazole has been identified as a direct inhibitor of the late endosomal/lysosomal cholesterol transport protein Niemann-Pick C1 (NPC1), thus severely disturbing the endosomal cholesterol balance [25,26,32]. The LE compartment is a critical host/pathogen interface in the IAV infection cycle, since the fusion of the IAV envelope with the LE limiting membrane is required for the transfer of the virus genome into the host cell cytoplasm, and ultimately into the cell nucleus. In our previous studies, we found that increased cholesterol accumulation via NPC1 blockade impacts IAV infection at multiple steps, (i) by impairing the virus/endosome fusion, (ii) by affecting the release and the infectivity of the virus progeny due to reduced cholesterol transport to the plasma membrane, and (iii) by priming the interferon response [24,25,26]. Our findings established the late endosomal cholesterol balance as a druggable cellular target for antiviral intervention. Importantly, we could confirm the therapeutic potential of a drug-repurposing approach in a preclinical mouse model of IAV infection [24]. The EC_50_ values for itraconazole-mediated antiviral activity against the two IAV subtypes tested on polarized Calu-3 cells in this study were very similar to the result obtained with another IAV strain in our earlier study, thus underscoring that itraconazole is indeed a promising candidate for the treatment of IAV infections [24].

Such drugs that target host processes required for pathogen replication and spread are considered to provide a novel means to limit infection. Because they impact basic cell functions, the development of viral resistance, as is frequently seen with drugs directly targeting the pathogen, such as the influenza virus NA inhibitor oseltamivir, is less likely [20,21]. Indeed, screening libraries of already approved compounds for novel uses (drug repurposing), has been established as a core drug development strategy, reducing the time and costs, due to the established safety profiles and known side effects of the tested compounds. Although drug repurposing for host-directed antiviral therapy is an approach that is considered a promising avenue, there are shortcomings to be considered, such as off-target and associated side effects on the host, as repurposed drugs often require high concentrations for their new use. Mechanistically, host-directed antiviral agents will slow down viral replication rather than eradicate the virus, and failure of HDT might result from the inappropriate time window of application (early vs. late infection stages) and a suppression rather than a complete elimination of the infectious agent, requiring long treatment duration which is often associated with poor patient compliance. Recently, combination therapies utilizing both host- and pathogen-directed strategies have been explored to overcome these limitations, as the combined use of two or more drugs might allow reduce the concentrations of the individual drugs in the cocktail [27,28,29]. As expected from established and clinically used drugs, no cytotoxic effects were observed when both, itraconazole and oseltamivir, were used individually and combined application reduced cell viability only at the highest concentrations tested. The pairwise combination was effective in reducing virus titers for both pdm09 and the Panama IAV variant. Oseltamivir and itraconazole EC_50_ values indicated that both drugs worked effectively and did not antagonize each other. This assumption was confirmed by Bliss independence drug interaction analysis, which pointed at independent drug actions [33]. Notably, we detected significantly decreased viral titers in the combination treatments when compared to monotherapy with oseltamivir, at lower drug concentrations than required for the single treatments. Moreover, the additional antiviral effect of itraconazole might function as a safeguard mechanism to combat IAV infection caused by strains with heightened oseltamivir resistance.

Clinical translation to exploit the effectiveness of a drug combination requires detailed knowledge of the pharmacokinetic drug profiles when given in combination, and novel combinations might be associated with hitherto unobserved side effects. Furthermore, the pharmacokinetic profiles need to be complementary in order to provide the required bioavailable doses at the same time. Transferring in vitro findings into clinical applications thus remains a challenge and might require the development of novel formulations and delivery approaches. This is especially relevant in the case of itraconazole, which has low a bioavailability after oral application [34,35,36]. Absorption from the gastrointestinal tract is limited because of the low water solubility of this very lipophilic compound. Low pH environments as gastric acid promote ionization and thus, absorption [34,35,37]. However, because the conversion into the soluble form is impaired by conditions impacting the gastric acidity such as fasting, chronic low gastric acidity, infections, and medications, effective itraconazole levels are difficult to adjust. Indeed, acidic beverages improve itraconazole absorption [38]. Results from our previous study exploring the benefit of itraconazole treatment in IAV-infected mice already documented the antiviral activity in vivo, and the results obtained in this study may help design trials in the treatment of influenza virus infections.

## References

[1] Miller M.S., Palese P. (2014). Peering into the crystal ball: Influenza pandemics and vaccine efficacy. Cell.

[2] Leang S.K., Deng Y.M., Shaw R., Caldwell N., Iannello P., Komadina N., Buchy P., Chittaganpitch M., Dwyer D.E., Fagan P. (2013). Influenza antiviral resistance in the Asia-Pacific region during 2011. Antivir. Res..

[3] Hurt A.C., Butler J., Kelso A., Barr I.G. (2012). Influenza antivirals and resistance: The next 10 years?. Expert Rev. Anti Infect. Ther..

[4] Carrat F., Flahault A. (2007). Influenza vaccine: The challenge of antigenic drift. Vaccine.

[5] Fauci A.S. (2006). Emerging and re-emerging infectious diseases: Influenza as a prototype of the host-pathogen balancing act. Cell.

[6] Villalón-Letelier F., Brooks A.G., Saunders P.M., Londrigan S.L., Reading P.C. (2017). Host cell restriction factors that limit influenza a infection. Viruses.

[7] Bouvier N.M., Palese P. (2008). The biology of influenza viruses. Vaccine.

[8] Yamauchi Y., Helenius A. (2013). Virus entry at a glance. J. Cell Sci..

[9] Edinger T.O., Pohl M.O., Stertz S. (2014). Entry of influenza A virus: Host factors and antiviral targets. J. Gen. Virol..

[10] Gasparini R., Amicizia D., Lai P.L., Bragazzi N.L., Panatto D. (2014). Compounds with anti-influenza activity: Present and future of strategies for the optimal treatment and management of influenza. Part I: Influenza life-cycle and currently available drugs. J. Prev. Med. Hyg..

[11] Lew W., Chen X., Kim C.U. (2000). Discovery and development of GS 4104 (oseltamivir): An orally active influenza neuraminidase inhibitor. Curr. Med. Chem..

[12] Kim S.G., Hwang Y.H., Shin Y.H., Kim S.W., Jung W.S., Kim S.M., Oh J.M., Lee N.Y., Kim M.J., Cho K.S. (2013). Occurrence and characterization of oseltamivir-resistant influenza virus in children between 2007–2008 and 2008–2009 seasons. Korean J. Pediatr..

[13] Jefferson T., Jones M.A., Doshi P., Del Mar C.B., Hama R., Thompson M.J., Spencer E.A., Onakpoya I.J., Mahtani K.R., Nunan D. (2014). Neuraminidase inhibitors for preventing and treating influenza in adults and children. Cochrane Database Syst. Rev..

[14] Liu S.S., Jiao X.Y., Wang S., Su W.Z., Jiang L.Z., Zhang X., Ke C.W., Xiong P. (2017). Susceptibility of influenza A(H1N1)/pdm2009, seasonal A(H3N2) and B viruses to Oseltamivir in Guangdong, China between 2009 and 2014. Sci. Rep..

[15] Mukherjee A., Nayak M.K., Dutta S., Panda S., Satpathi B.R., Chawla-Sarkar M. (2016). Genetic characterization of circulating 2015 A(H1N1)pdm09 influenza viruses from Eastern India. PLoS ONE.

[16] Hayden F.G., Sugaya N., Hirotsu N., Lee N., de Jong M.D., Hurt A.C., Ishida T., Sekino H., Yamada K., Portsmouth S. (2018). Baloxavir Marboxil for Uncomplicated Influenza in Adults and Adolescents. N. Engl. J. Med..

[17] Hu Y., Musharrafieh R., Ma C., Zhang J., Smee D.F., DeGrado W.F., Wang J. (2017). An M2-V27A channel blocker demonstrates potent in vitro and in vivo antiviral activities against amantadine-sensitive and -resistant influenza A viruses. Antivir. Res..

[18] He G., Qiao J., Dong C., He C., Zhao L., Tian Y. (2008). Amantadine-resistance among H5N1 avian influenza viruses isolated in Northern China. Antivir. Res..

[19] Shaw M.L. (2011). The host interactome of influenza virus presents new potential targets for antiviral drugs. Rev. Med. Virol..

[20] Basler C.F. (2007). Influenza viruses: Basic biology and potential drug targets. Infect. Disord. Drug Targets.

[21] de Chassey B., Meyniel-Schicklin L., Vonderscher J., André P., Lotteau V. (2014). Virus-host interactomics: New insights and opportunities for antiviral drug discovery. Genome Med..

[22] Kaufmann S.H.E., Dorhoi A., Hotchkiss R.S., Bartenschlager R. (2018). Host-directed therapies for bacterial and viral infections. Nat. Rev. Drug Discov..

[23] Viasus D., Paño-Pardo J.R., Cordero E., Campins A., López-Medrano F., Villoslada A., Fariñas M.C., Moreno A., Rodríguez-Baño J., Oteo J.A. (2011). Effect of immunomodulatory therapies in patients with pandemic influenza A (H1N1) 2009 complicated by pneumonia. J. Infect..

[24] Schloer S., Goretzko J., Kühnl A., Brunotte L., Ludwig S., Rescher U. (2019). The clinically licensed antifungal drug itraconazole inhibits influenza virus in vitro and in vivo. Emerg. Microbes Infect..

[25] Kühnl A., Musiol A., Heitzig N., Johnson D.E., Ehrhardt C., Grewal T., Gerke V., Ludwig S., Rescher U. (2018). Late endosomal/lysosomal cholesterol accumulation is a host cell-protective mechanism inhibiting endosomal escape of influenza A virus. MBio.

[26] Musiol A., Gran S., Ehrhardt C., Ludwig S., Grewal T., Gerke V., Rescher U. (2013). Annexin A6-balanced late endosomal cholesterol controls influenza a replication and propagation. MBio.

[27] Melville K., Rodriguez T., Dobrovolny H.M. (2018). Investigating different mechanisms of action in combination therapy for influenza. Front. Pharmacol..

[28] Dunning J., Baillie J.K., Cao B., Hayden F.G. (2014). Antiviral combinations for severe influenza. Lancet Infect. Dis..

[29] Popov A.F., Shchelkanov M.Y.E. (2018). Combined Therapy of Influenza with Antiviral Drugs with a Different Mechanism of Action in Comparison with Monotherapy. J. Pharm. Sci. Res..

[30] Faul F., Erdfelder E., Lang A.G., Buchner A. (2007). G*Power 3: A flexible statistical power analysis program for the social, behavioral, and biomedical sciences. Proceedings of the Behavior Research Methods.

[31] Ianevski A., Giri A.K., Aittokallio T. (2020). SynergyFinder 2.0: Visual analytics of multi-drug combination synergies. Nucleic Acids Res..

[32] Trinh M.N., Lu F., Li X., Das A., Liang Q., De Brabander J.K., Brown M.S., Goldstein J.L., Chang T.-Y., Maxfield F.R. (2017). Triazoles inhibit cholesterol export from lysosomes by binding to NPC1. Proc. Natl. Acad. Sci. USA.

[33] Liu Q., Yin X., Languino L.R., Altieri D.C. (2018). Evaluation of Drug Combination Effect Using a Bliss Independence Dose–Response Surface Model. Stat. Biopharm. Res..

[34] Hurlé A.D.-G., Navarro A.S., Sánchez M.J.G. (2006). Therapeutic drug monitoring of itraconazole and the relevance of pharmacokinetic interactions. Clin. Microbiol. Infect..

[35] Shin J.H., Choi K.Y., Kim Y.C., Lee M.G. (2004). Dose-Dependent Pharmacokinetics of Itraconazole after Intravenous or Oral Administration to Rats: Intestinal First-Pass Effect. Antimicrob. Agents Chemother..

[36] Allegra S., Fatiguso G., De Francia S., Favata F., Pirro E., Carcieri C., De Nicolò A., Cusato J., Di Perri G., D’Avolio A. (2017). Pharmacokinetic evaluation of oral itraconazole for antifungal prophylaxis in children. Clin. Exp. Pharmacol. Physiol..

[37] Lestner J., Hope W.W. (2013). Itraconazole: An update on pharmacology and clinical use for treatment of invasive and allergic fungal infections. Expert Opin. Drug Metab. Toxicol..

[38] Bae S.K., Park S.-J., Shim E.-J., Mun J.-H., Kim E.-Y., Shin J.-G., Shon J.-H. (2011). Increased Oral Bioavailability of Itraconazole and Its Active Metabolite, 7-Hydroxyitraconazole, When Coadministered With a Vitamin C Beverage in Healthy Participants. J. Clin. Pharmacol..

